# Tolerance induction through early feeding to prevent food allergy in infants with eczema (TEFFA): rationale, study design, and methods of a randomized controlled trial

**DOI:** 10.1186/s13063-022-06126-x

**Published:** 2022-03-12

**Authors:** Birgit Kalb, Lara Meixner, Valérie Trendelenburg, Nathalie Unterleider, Josefine Dobbertin-Welsch, Stephanie Heller, Sabine Dölle-Bierke, Stephanie Roll, Susanne Lau, Young-Ae Lee, Florent Fauchère, Julian Braun, Magda Babina, Sabine Altrichter, Till Birkner, Margitta Worm, Kirsten Beyer

**Affiliations:** 1 Department of Pediatric Respiratory Medicine, Immunology and Critical Care Medicine, Charité – Universitätsmedizin Berlin, corporate member of Freie Universität Berlin and Humboldt-Universität zu Berlin, Augustenburgplatz 1, 13353 Berlin, Germany; 2 Division of Allergy and Immunology, Department of Dermatology, Venerology and Allergy, Charité – Universitätsmedizin Berlin, corporate member of Freie Universität Berlin and Humboldt-Universität zu Berlin, Berlin, Germany; 3 Institute of Epidemiology, Social Medicine and Health Economics, Charité – Universitätsmedizin Berlin, corporate member of Freie Universität Berlin and Humboldt-Universität zu Berlin, Berlin, Germany; 4 Max Delbrück Center For Molecular Medicine in the Helmholtz Association, Charité – Universitätsmedizin Berlin, corporate member of Freie Universität Berlin and Humboldt-Universität zu Berlin, Berlin, Germany; 5 Berlin Institute of Health at Charité - Universitätsmedizin Berlin, BIH Center for Regenerative Therapies, Berlin (BCRT), Berlin, Germany; 6 Si-M/“Der Simulierte Mensch” a science framework of Technische Universität Berlin and Charité - Universitätsmedizin Berlin, Berlin, Germany; 7 Division of Dermatological Allergy, Department of Dermatology, Venerology and Allergy, Charité – Universitätsmedizin Berlin, corporate member of Freie Universität Berlin and Humboldt-Universität zu Berlin, Berlin, Germany; 8 Universitätsklinikum für Dermatologie und Venerologie, Kepler Uniklinikum Linz, Linz, Austria; 9 Experimental and Clinical Research Center, a cooperation between the Max-Delbrück-Center for Molecular Medicine in the Helmholtz Association and the Charité - Universitätsmedizin Berlinz, Berlin, Germany; 10 Charité – Universitätsmedizin Berlin, corporate member of Freie Universität Berlin and Humboldt-Universität zu Berlin, Experimental and Clinical Research Center, Lindenberger Weg 80, 13125 Berlin, Germany

**Keywords:** Protocol, Randomized controlled trial, Food allergy, Eczema, Atopic dermatitis, Prevention, Early feeding, Weaning foods

## Abstract

**Background:**

Up to 8% of all children in industrialized countries suffer from food allergies, whereas children with atopic eczema are affected considerably more frequently. In addition, the type and starting time of weaning foods seem to influence the development of food allergies. However, data from interventional studies on weaning are controversial.

The aim of this randomized-controlled clinical trial is to investigate, whether an early introduction of hen's egg (HE), cow’s milk (CM), peanut (PN), and hazelnut (HN) in children with atopic eczema can reduce the risk for developing food allergies in the first year of life.

**Methods:**

This is a protocol for a randomized, placebo controlled, double blind, single-center clinical trial.

One hundred fifty infants with atopic eczema at 4–8 months of age will be randomized in a 2:1 manner into an active group that will receive rusk-like biscuit powder with HE, CM, PN, and HN (initially approximately 2 mg of each food protein) for 6–8 months or a placebo group, whose participants will receive the same rusk-like biscuit powder without HE, CM, PN, and HN on a daily basis. During the interventional period, the amount of allergens in the study product will be increased three times, each after 6 weeks. All study participants who are sensitized to HE, CM, PN, or HN at the end of the interventional period will undergo an oral food challenge to the respective food in a further visit.

Primary endpoint is IgE-mediated food allergy to at least one of the four foods (HE, CM, PN or HN) after 6–8 months of intervention (i.e., at around 1 year of age). Secondary endpoints include multiple food allergies, severity of eczema, wheezing, and sensitization levels against food allergens.

**Discussion:**

This clinical trial will assess whether an early introduction of allergenic foods into the diet of children with atopic eczema can prevent the development of food allergies. This trial will contribute to update food allergy prevention guidelines.

**Trial registration:**

German Clinical Trials Register DRKS00016770. Registered on 09 January 2020.

**Supplementary Information:**

The online version contains supplementary material available at 10.1186/s13063-022-06126-x.

## Background

IgE-mediated food allergy is a burden in industrialized countries with up to 8% of all infants and children suffering from this condition [1]. Especially infants with eczema are at high risk for developing food allergies [2]. About half of these children become sensitized and about one third will develop clinically relevant food allergy [1, 3, 4]. The main elicitors are HE, CM, PN and tree nuts. Food allergy often starts in infancy and interestingly the children become sensitized against certain foods even before eating them for the first time. It is the current understanding that sensitization occurs via the cutaneous route due to an impaired skin barrier function in these infants [2]. In the past, prevention strategies concerning the development of food allergy have been driven by avoidance of allergenic foods in high-risk infants. Despite these attempts, a rising prevalence of food allergy has been observed [1, 5]. Therefore, current guidelines no longer recommend avoidance [6, 7]. However, the optimal way of allergen introduction into the infants´ diet is still under debate. The main questions are “when,” “how,” “which allergen,” and “which children.”

To address these questions, several randomized controlled studies have been conducted with different foods [8–14]. The LEAP trial in the UK focused on peanut allergy in high-risk children (severe eczema and/or hen’s egg allergy) [8]. After randomization, 540 participants from 4 to 11 months of age were asked to consume peanut three times weekly or to avoid peanut strictly until the age of 5 years. In this high risk population, early and continuous feeding of peanut products reduced the development of peanut allergy by 80% [8]. However, it is still unclear how these results can be transferred to other countries where peanuts are less frequently consumed. For instance, data from Singapore showed very low prevalence rates of peanut allergy despite the fact that peanuts are not introduced into infants’ diets in the first year of life [9].

Regarding HE, five intervention studies showed no clear reduction of allergy but high rates of adverse reactions, both immediate type symptoms and gastrointestinal problems [10–12]. However, one small study from Japan showed a significant reduction of hen’s egg allergy without adverse events. In contrast to the other prevention studies, they started with very small amounts of heated egg powder, increasing to small amounts after 3 months. One hundred twenty-one infants were enrolled at 4–5 months of age with two thirds of the infants being already sensitized to hen’s egg with unknown clinical relevance. At 12 months of age, only 8% of the active group versus 38% of the placebo group had developed hen’s egg allergy [13].

Many children develop food allergy to more than one allergen and prevention strategies should keep this in mind. Another randomized but not placebo-controlled trial in the UK, the EAT study, focused on the effects of the early administration of various foods to breast-fed infants from the general population [14]. From a total of 1303 children, in an intervention group, six different allergenic foods were introduced into their diet, starting at 3 months of age (early introduction group), while the standard introduction group was exclusively breast-fed until 6 months of age. In the per-protocol analysis, the prevalence of any food allergy was significantly lower in the early introduction group than in the standard introduction group (2.4% versus 7.3%). However, in the intention-to-treat-analysis, there was no statistically significant difference between both groups (5.6% versus 7.1%), because in the majority of infants, the protocol was not followed [14]. Reasons for this might be the complicated protocol of consecutive food introduction, or adverse reactions.

In conclusion, further research is needed before evidence-based advice can be given for the prevention of food allergy.

Therefore, we will introduce small amounts of four highly allergenic foods (baked HE, CM, PN, and HN) together in infants with eczema at 4–8 months of age in a randomized, placebo-controlled, clinical trial in order to assess oral tolerance development. This is an abridged protocol based on protocol version 4.0 dated 05 April 2021. The full protocol is available on: German clinical trials register http://www.drks.de (DRKS00016770 or https://www.drks.de/drks_web/navigate.do?navigationId=trial.HTML&TRIAL_ID=DRKS00016770).

This protocol adheres to the Standard Protocol Items: Recommendations for Interventional Trials (SPIRIT) recommendations for interventional trials and the SPIRIT Checklist is included (see Additional file 1) [15].

## Methods

### Objectives

The primary objective is to assess the efficacy (superiority) of an early introduction of small amounts of allergenic foods on the development of food allergy in the first year of life in infants with eczema. Secondary objectives are to investigate if an early introduction of small amounts of allergenic foods in the first year of life is safe or if it results in the occurrence of allergic reactions, gastrointestinal problems, or in failure to thrive. Moreover, the impact of an early introduction of small amounts of allergenic foods on the development of multiple food allergies, severity of eczema, the prevalence of asthma, and distinct immunoglobulin pattern in the first year of life will be assessed.

### Trial design

**Fig. 1:**
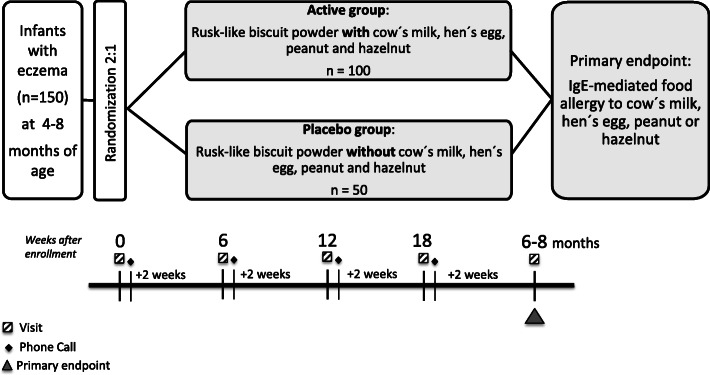
Study design overview. The diagram shows the flow of the patients after enrolment and randomization in the active or the placebo group. Subjects in the active group will receive a sugar-free rusk-like biscuit powder with hen´s egg, cow’s milk, peanut, and hazelnut, while subjects in the placebo group will receive a sugar-free rusk-like biscuit powder without these allergens. After 6 months of intervention, we will check for sensitization against hen’s egg, cow’s milk, hazelnut, and peanut. All sensitized subjects will receive the study product until they will undergo an oral food challenge against the corresponding foods, while in the non-sensitized infants the intervention will be stopped

Tolerance induction through Early Feeding to prevent Food Allergy in infants with eczema (TEFFA) is a randomized, placebo controlled, double blind, single center trial with two parallel groups (Fig. 1). All study participants who are sensitized to HE, CM, PN, or HN at the end of the interventional period will undergo an oral food challenge (OFC) to the respective food after 6–8 months.

TEFFA is part of the clinical research unit (CRU) 339 “food allergy and tolerance” (FOOD@). Using biological samples obtained within the trial, several mechanistic sub-projects will be performed in order to gain a deeper understanding of the immunological mechanisms involved in food allergy and tolerance development. In particular, the role of the gastrointestinal as well as the skin microbiome, the IgEome, epigenetic mechanisms, antigen specific immunologic reactivities as well as inflammatory circuits, and serological biomarkers will be investigated. The methods of these sub-projects will be described elsewhere.

### Setting

This single-center trial is recruiting participants at the Charité - Universitätsmedizin and at regular well-baby check-ups at primary care physicians in Berlin. Additionally, we aim to approach participants via flyer advertisement, advertisement in journals, magazines, and social media as well as at information events for (prospective) parents. All visits throughout the study will take place either in the study center (V1-V5) or on ward (V6: DBPCFC).

### Participants

Infants with eczema 4–8 months of age are eligible for the trial. Eczema is defined as doctor´s diagnosed or parent-reported doctor´s diagnosed and will be finally evaluated by the study physician according to the criteria of Hanifin and Rajka [16]. Infants are excluded from the study if they have previously consumed HE, PN, or HN. Previous consumption of CM is no exclusion criterion, since infant formula commonly is based on CM and formula fed infants should be also addressed. Further exclusion criteria are wheat allergy (study product contains wheat), health issue (cardiac or congenital), and participation in another interventional trial.

### Consent procedure

All legal representatives of the participants must read, sign, and date the informed consent form before entering the study or undergoing any study-specific procedures. Before consent is given, the investigator or his/her representative will explain verbally the aim, method, source of funding, and the anticipated benefits and potential risks of the study to the parents, answer all questions regarding the study, and document the informed consent process. As opt-it consent, included in the main study consent form, biological specimens are collected and partially stored for future analysis. A unique participant number will be allocated to each participant and assigned chronologically prior to proceeding with study screening. The sequential identification numbers rather than names will be used to collect, store, and report participant information.

### Intervention

In the active group, subjects consume a sugar-free, rusk-like biscuit powder daily, which contains small amounts of HE, CM, PN, and HN. The allergen doses in the study product will be approximately 2 mg of each food protein at 4–8 months of age and will be increased three times during the study every 6 weeks. The last and highest dosing step will be fed until the individual end of the study. Taking both safety and efficacy into account, the initial dose of allergens in the study powder as well as the respective up-dosings were determined on the basis of ED_05_ [17]. The target allergen doses for each study period were verified by analyzing the final study product. Table 1 summarizes the results obtained within this analysis.

**Table 1:** Allergen amount in study product (amount of daily protein)

	Amount of protein daily [mg]			
	Peanut	Hazelnut	Hen’s egg	Cow´s milk
**Active group**				
**Dose 1 (V1–V2)**	1.7	1.9	1.8	2.0
**Dose 2 (V2–V3)**	8.4	10.0	9.0	10.0
**Dose 3 (V3–V4)**	39	44	42	45
**Dose 4 (V4–V5/V6)**	146	164	155	167
**Placebo group**				
**V1–V5/V6**	0	0	0	0

The sugar-free, rusk-like biscuit powder (3 g of powder/day using a measuring spoon) will be mixed with water, and optionally fruits, to a mash. The study product should be eaten on a daily basis throughout the study duration. The first feeding of the study product as well as every dose increase, each after 6 weeks, will be performed under medical supervision at the study center. In case of adverse events, the intervention may be individually modified according to investigator’s discretion.

In the placebo group, subjects consume daily a sugar-free rusk-like biscuit powder, without HE, CM, PN, and HN, analogously to the intervention group. The first and next dosing step feedings of the placebo study product will also be performed under medical supervision at the study site to maintain the blinding.

The first weaning food (e.g., vegetables) should have been successfully introduced for at least 1 week before feeding of the active/placebo product. Mothers will be encouraged to continue with breastfeeding while introducing weaning foods. Parents of both groups will be advised to avoid HE, PN, and HN or their products in their child’s diet. If the subject is unable to consume the recommended amount of rusk like biscuit powder, i.e., in the frame of feeding difficulties when weaning foods are introduced, the investigator and study physician may adjust the amount or dosing regimen within the patient’s diet. Non-compliance is defined as a repeated event of insufficient consumption of the intervention product, i.e., less than 3 times/week for 3 weeks (either during 3 consecutive weeks or three repeated events of 1 week). All participants may continue their usual medications, also those taken for any concomitant diseases, including wheezing and eczema, throughout the study. All subjects who will undergo an OFC at visit 6 will be advised to discontinue oral antihistamines 3–5 days before this procedure.

### Primary endpoint

The primary endpoint is the occurrence of IgE-mediated food allergy determined with level 1 or 2 to at least one of the four foods (HE, CM, PN or HN) after 6–8 months of intervention (at around 1 year of age).

The diagnosis of food allergy will be determined according to the following levels:

- Level 1: A positive oral food challenge at around one year of age in a child with allergen-specific IgE > 0.1 kU/l or skin prick test (SPT) wheel size > 3 mm.
- Level 2: A systemic reaction with objective symptoms upon accidental exposure in a child with specific IgE to one of the four intervention foods > 0.1 kU/l or SPT wheel size > 3 mm.
- Level 3: Allergen- or allergen component-specific IgE above the 90% predicted probability for this individual food [18, 19].

### Secondary endpoints

- After 6–8 months of intervention (at around 1 year of age):
  - IgE-mediated food allergy determined with level 1 to at least one of the four foods HE, CM, PN, and HN
  - IgE-mediated food allergy determined with level 1, 2 or 3 to at least one of the four foods HE, CM, PN, and HN
  - IgE-mediated food allergy determined with level 1 or 2 to at least one of the three foods HE, PN, and HN
  - IgE-mediated food allergy determined with level 1 or 2 to HE
  - IgE-mediated food allergy determined with level 1 or 2 to CM
  - IgE-mediated food allergy determined with level 1 or 2 to PN
  - IgE-mediated food allergy determined with level 1 or 2 to HN
  - IgE-mediated food allergy determined with level 1, 2, or 3 separately to HE
  - IgE-mediated food allergy determined with level 1, 2, or 3 separately to CM
  - IgE-mediated food allergy determined with level 1, 2, or 3 separately to PN
  - IgE-mediated food allergy determined with level 1, 2, or 3 separately to HN
  - Number of food allergies (2, 3, 4, 5, > 6)
- Occurrence (frequency and severity) of immediate type allergic symptoms during the whole study period
- Occurrence of gastrointestinal symptoms during the whole study period
- Growth during the whole study period
- Change of eczema status (measured by SCORAD (Scoring atopic dermatitis)) after 6, 12, and 18 weeks and 6 months of intervention and at oral food challenge in those subjects who are sensitized against food allergens after 6 months of intervention
- Change of eczema status (measured by EASI score (Eczema Area and Severity Index)) after 6, 12, and 18 weeks and 6 months of intervention and at oral food challenge in those subjects who are sensitized against food allergens after 6 months of intervention
- Change in topical eczema treatment after 6, 12, and 18 weeks and 6 months of intervention and at oral food challenge in those subjects who are sensitized against food allergens after 6 months of intervention
- Wheezing after 6, 12, and 18 weeks and 6 months of intervention and at oral food challenge in those subjects who are sensitized against food allergens after 6 months of intervention
- Allergen-specific IgE and IgG4 to HE, CM, PN, HN, Ara h2, Cor a14, and wheat after 6 months of intervention (at around 1 year of age)

### Randomization, allocation concealment, and blinding

After enrolment, participants will be randomly assigned to the active or placebo group in a 2:1 manner. The randomization will be prepared by the trial statistician and will be implemented within the REDCap (Research Electronic Data Capture) database system by the data manager. The randomization will include a technical stratification regarding singleton/twin status. Singletons will be assigned to the treatment groups by simple randomization in a 2:1 manner (active: placebo). Twins will also be randomly assigned to the treatment groups in a 2:1 manner (active: placebo); however, twins will be randomized together, i.e., each pair will be randomized into the same treatment group. Thus, the ratio of 2:1 will be implemented on the pair-level. In addition, the randomization of twins will be blocked (block length: 3 pairs (i.e., 6 subjects)) due to the low number of twins expected in this study. The rationale for randomizing twins into the same treatment group is the difficulty for parents to ensure the strict separation of the daily (blinded) feeding products to their infants over the course of 6–8 months. Study investigators, all involved study staff, and the parents of the participants are blinded to treatment. Allocation of subjects will be performed concealed (i.e., without knowledge of group allocation) by authorized site personnel using REDCap where each participant will be allocated to one out of fifteen kit codes for the interventional product (10 codes active, 5 codes placebo). The product will be labeled with the kit codes by staff, which is unblinded, but not otherwise involved in the study.

In general, unblinding per subject will be performed at the individual end of the study (if the patients are sensitized to HE, CM, PN, or HN at V5, they are instructed to continue consuming the study product until V6 takes place.) In case of serious allergic reactions suspected to be related to the study intervention, unblinding should be performed on the decision of the investigator. In order to ensure the unblinding process, emergency envelopes, providing information on the individual interventional group the subject is assigned to, are stored at the study center. Once unblinding has been performed, it will be documented in the source data, reported to the principal investigator and will result in the subjects’ early discontinuation of intervention.

### Study procedures

Screening (visit 1, V1) of the subjects will take place at 4–8 months of age in infants who developed eczema. Written informed consent will be obtained from the parents by the study physician. During V1, the inclusion and exclusion criteria will be checked. Subjects who are eligible for participation in the study will be randomized to one of the two study groups. Information on demographics, subject/family characteristics, relevant medical history, medication, and nutrition will be recorded. In addition, anthropometric measurements and physical examination will be performed, including the assessment of eczema severity using SCORAD [20] and EASI score [21]. Blood from the participant and its mother will be drawn. Skin swabs and saliva samples will be collected for the mechanistic subprojects by the study staff. Skin swabs are collected and preserved in DNA/RNA shield collection tubes containing medium (DNA/RNA Shield; Zymo Research, Irvine, CA, USA). Saliva samples are collected both with Zymo Swabs as well as with Salimetric Swabs (SalivaBio Swab; Suffolk, Great Britain). Transepidermal water (TEWL) measurement will be performed with the Tewameter TM Hex (Courage + Khazaka electronic GmbH, Germany). Palmar linearity pattern will be analyzed and documented by palm photographs.

The investigator will instruct the parents to collect stool samples (using OMNIgene-GUT tubes, OMR-200; DNA Genotek, Ontario, Canada) from the infants at home for the mechanistic subprojects. The parents should further collect a dust sample (using dustream® collector DU-ST-1; Indoor Biotechnologies LTD, Cardiff, UK) from the infant´s bed (or the parent´s bed, if the child sleeps in this bed four nights or more per week) and the living room.

The first feeding of the study product will be performed at the study site. Parents will be asked to maintain an e-diary from V1 to V5/V6. The e-diary contains questions concerning the patient´s diet, compliance, and tolerability of the study product, eczema severity (POEM score [22]) and concomitant medication.

Supplies of study product will be dispensed at each visit. Parents will be instructed to contact the study site in case of an immediate type allergic reaction after food exposure (either to the study product or due to exposure to any food allergens). Additionally, in case of recurrent gastrointestinal symptoms, worsening of eczema or any suspected adverse event parents are asked to contact the study site. Two weeks after V1, parents will be called (phone call 1; PC1) to investigate compliance and tolerability of the study product and to promote retention. Subjects will be required to visit the study site after 6 (V2), 12 (V3) and 18 (V4) weeks, and 6 (V5) months of intervention. At V2, V3, V4, and V5, information on medical history, medication (especially treatment of eczema), and nutrition (breast feeding, complementary foods) will be recorded. In addition, anthropometric measurements and a physical examination including SCORAD and EASI score will be performed. Stool, dust, skin swabs, and saliva samples will be collected. TEWL will be performed. At V2, V3, and V4, the first feeding of the next higher dose of the verum, respectively of the placebo in order to ensure blinding, will be performed at the study site. Supplies of study product will be dispensed at each visit. Two weeks after V2, V3, and V4, parents will be called (PC2, PC3, and PC4) to investigate compliance and tolerability of the study product. An additional blood sample from the participant and its mother will be collected, and a SPT with HE, CM, PN, and HN will be performed at V5 (all extracts including positive and negative controls: ALK Abelló, Germany). Participants sensitized to any of the checked foods (elevated food-specific IgE (> 0.1 kU/l) or positive SPT (wheel size > 3 mm)) will be invited to undergo an OFC at V6. Sensitized patients are instructed to continue consuming the study product until V6 will take place. OFC will be performed in a double-blinded, placebo-controlled manner in accordance with clinical routine practice of the Charité-Universitätsmedizin Berlin, based on PRACTALL international guidelines for OFC and stopped using standardized stopping criteria based on PRACTALL guidelines [23]. Roasted, defatted peanut flour, defatted HN flour, fresh CM, and pasteurized raw HE will be used for OFC. Up to seven increasing dose steps at 30-min intervals using a semi log scale ranging approximately from 2 mg to 3 g food protein (depending on the individual allergen) will be administered. In case of absence of any objective, allergic symptoms, a cumulative dose up to 4.5 g food protein will be administered on another day. In patients with allergic reactions to raw HE and fresh CM, an additional food challenge will be offered with baked HE or CM, respectively. Participants who developed a clinical relevant food allergy at the end of the trial will receive an individualized dietary counseling. Moreover, parents are offered to contact the outpatient clinic of the Department of Pediatric Pulmonology, Immunology and Critical Care Medicine, Charité – Universitätsmedizin Berlin, for further consultation. In case of discontinuation or deviation from the intervention protocol, all participants are encouraged to maintain the scheduled visits or phone calls and collection of all possible data is planned. For detailed description of all study procedures, see Table 2.

**Table 2:**
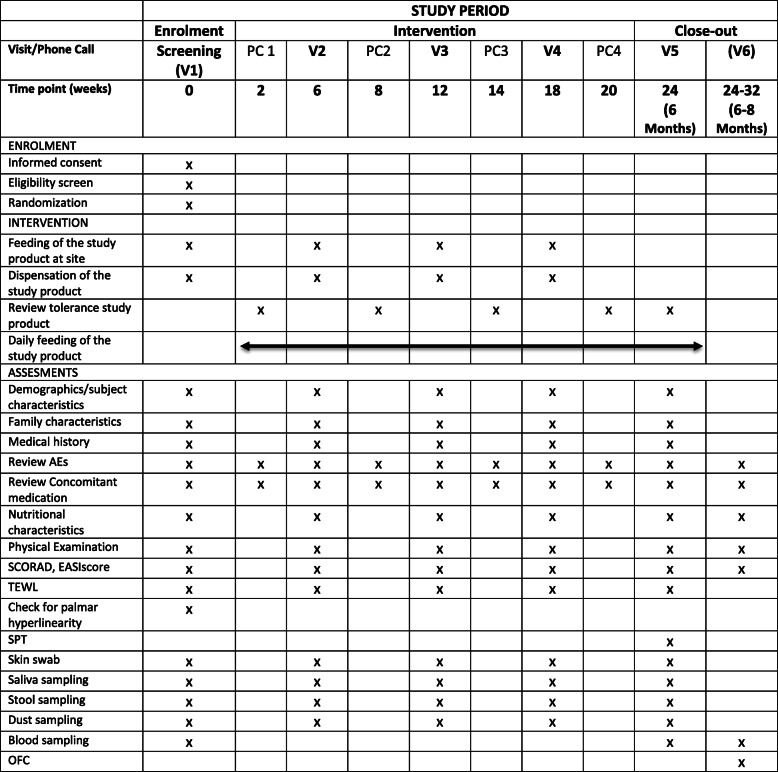
Study visits and procedures

*Abbreviations*: *V* visit, *PC* phone call, *AE* adverse event, *TEWL* transepidermal water loss measurement, *SPT* skin prick testing, *OFC* oral food challenge

### Data collection and management

REDCap (Research Electronic Data Capture) will be used as electronic case report form (eCRF) to collect and manage study data. REDCap is hosted at the Charité – Universitätsmedizin Berlin and provides an interface for data entry for clinicians. In addition, it provides a data integration platform for the data from the mechanistic subprojects. Audit-trails will be integrated for tracking data entries, corrections, and import/export procedures. A unique participant number will be assigned to every screened patient consisting with a prefix and a consecutive numbering. This unique code will identify all patient-specific data, e.g., clinical study data as well as biosample results. Any adverse event will be documented on paper documents (diary). Adverse events of interest (recurrent gastrointestinal symptoms, worsening of eczema and allergic reactions) as well as serious adverse events (SAEs) will be additionally documented in the REDCap eCRF. In case of any adverse of interest, parents are advised to contact the study site as soon as possible. SAEs have to be reported within 48 h after knowledge of event to the principal investigator. Data access and storage will follow the data security concept of the Charité including password-protected access to all computers and folders, which contain sensitive data. Pseudonymized data will be made available to all groups participating in the CRU.

### Sample size calculation

Analyzing 120 infants (80 in the active group and 40 in the placebo group) will result in at least 80% power to detect a difference in the primary endpoint (food allergy after 6–8 months of intervention) of 10% in the intervention group vs. 33% in the control group, based on a two-sided chi-squared test with significance level 5%. In order to compensate for a potential dropout-rate of about 20%, 150 infants will be randomized (100 infants to the active group and 50 infants to the placebo group). As the number of twins included to this study is expected to be very low (i.e., one or two pairs), the correlation due to twin status is not taken into account for the sample size calculation.

### Statistical analysis

The primary analysis of the primary endpoint will be performed by logistic regression with (fixed) factor treatment group (active, placebo) taking the correlation of twins into account. From this model, the odds ratio for treatment will be calculated with 95% confidence interval and a *p*-value for the treatment group comparison. The analysis will be performed on the full analysis set (FAS) without imputation of missing values. The significance level will be set to 0.05 (two-sided). All other analysis will be considered explorative. Several explorative sensitivity analysis will be performed for the primary endpoint: a re-run of the primary analysis model without taking the correlation of twins into account and a re-run of the primary analysis model with the per- protocol population (instead of the FAS). In case of relevant differences between the treatment groups with respect to baseline variables, the primary analysis will be repeated with further adjustment variables (FAS population). Moreover, several subgroup analysis will be conducted regarding the following subgroups: carrier of mutations in the Filaggrin gene; infants with at least one parent with eczema; infants with at least one parent with atopic diseases (asthma, rhinoconjunctivitis, eczema); breastfed children/formula fed children, children with impaired skin barrier function (measured with TEWL); and household allergen exposure. Subgroup analysis will be performed by additionally including the subgroup variable and the interaction term (subgroup*treatment group) into the primary analysis model (FAS population). In case subpopulations regarding to the consumption of CM exist, these subpopulations will be analyzed separately (FAS population).

Explorative analysis of secondary endpoints will follow the same principle as the primary analysis of the primary endpoint, i.e., models with (fixed) factor treatment group, taking the correlation of twins into account. Binary endpoints will be analyzed by logistic regression; continuous endpoints will be analyzed by analysis of covariance (ANCOVA) with the respective baseline value (if available) as additional covariate. Secondary endpoints will be analyzed with the FAS; selected secondary endpoints will also be analyzed with the per-protocol population. Concerning safety endpoints, the nature, frequency, and severity of adverse events and safety variables, including serious adverse events, will be summarized descriptively by treatment group. As a general strategy, missing data will not be imputed in this study. No interim analyses for efficacy are planned.

## Discussion

This clinical trial, including 150 infants with eczema at 4–8 months of age, will investigate whether an early introduction of baked HE, CM, PN, and HN can reduce the risk for developing food allergy in the first year of life.

The study population of this trial consists of infants with eczema, who are at risk for development of further atopic conditions, and therefore does not represent the general population. Interestingly, it has been reported that even in children with food allergy but without eczema, an impaired skin barrier function occurs [24]. One third of the infants taking part in our HEAP study, who were sensitized against HE, had no atopic dermatitis at the screening visit [10]. Nevertheless, Natsume et al. were able to show that the introduction of small amounts of heated HE is particularly effective in infants with atopic dermatitis that might already be sensitized [13]. It needs to be shown, whether this finding can be confirmed and whether this approach is suitable for other food allergens. Besides the introduction of HE, Natsume et al. treated participants’ eczema attentively throughout the whole study period. In our trial, the treatment of the subjects’ eczema will not be part of the study intervention. However, during the interventional period of our study treatment of eczema will be assessed and the impact of an early introduction of small amounts of allergenic foods in regard to severity of eczema will be evaluated. Severity grading will be performed by using different objective and standardized diagnostic systems (POEM, SCORAD, and EASI diagnostic score).

Since many children seem to develop food allergy to more than one food [14, 25], a further strength of our study is that we introduce multiple food allergens at the same time, while keeping the study procedures for the participants as simple as possible. Although it was not feasible to integrate all common food allergens in our interventional product, it does contain the most important ones (HE, CM, PN, and HN). Recently, Skjerven et al. evaluated the early and regular use of skin care and the early complementary feeding of PN, CM, HE, and wheat (WT) at 3–4 months of age in the general infant population. Participants were randomized to either a skin intervention, a food intervention, a combined intervention or a no intervention group. The food intervention group was instructed to introduce PN at 3–4 months of age, followed by CM 1 week later, WT the next week, and finally HE in the fourth week. The infants´ parents were advised to administer each of the foods from the finger or from a teaspoon at least 4 days per week. Although the final results have not been published yet, Skjerven and colleagues reported that the adherence to the food intervention was quite low (32%) [26]. This observation and the results of the EAT study underline that the introduction of food allergens has to be suitable for real life settings at this very young age, in order to guarantee compliance [14, 26].

In case of sensitization against certain foods, determined at the end of the interventional period, OFC will be performed in a double-blinded, placebo-controlled manner, representing the gold standard procedure in diagnosing food allergy.

Within this study, one of our aims is to assess the safety of an early introduction of small amounts of allergenic foods in infants with eczema in the first year of life regarding the occurrence of immediate type allergic reactions, gastrointestinal symptoms, and growth. Currently, children with moderate to severe eczema are routinely screened for sensitization against the most important food allergens [27]. In order to avoid anaphylactic reactions occurring at home within the first feeding, screening for sensitization against certain food allergens needs to be conducted before these foods are introduced into the infant´s diet [27]. The results of this trial will help to clarify, if an early introduction of potentially allergenic foods in high-risk infants can be done safely by starting from a very small dose and increasing the amount in a stepwise manner, even without screening for sensitization before.

The results of the trial will be submitted for publication in peer-reviewed journals and will further be communicated to participants, health care providers, and professional groups including health visitors and midwives, as they may affect current dietary recommendations regarding the prevention of food allergy.

## Trial status

This is an abridged protocol based on protocol version 4.0 dated 05 April 2021.

The first patient was randomized in February 2020, and the last patient is planned to be enrolled in February 2023.

## Supplementary Information

**Additional file 1.** The SPIRIT Checklist

## Data Availability

All data generated in the CRU will be curated and organized into a set of files, shared on an online, publicly available data repository after peer-reviewed publication (preservation and accessibility of the data) to ensure potential secondary analyses, long-term archiving, and reuse by other researchers (scientific recognition). Besides the study protocol, publications are planned for the results in peer-reviewed journals. In addition, results will be communicated in lay language to participants and health care providers as they may affect current food prevention guidelines.

## Abbreviations

AE: Adverse event
ANCOVA: Analysis of covariance
BIH: Berlin Institute of Health
CRF: Case report form
CRU: Clinical research unit
DBPCFC: Double-blind placebo-controlled food challenge
DFG: German Research Foundation
EASI-Score: Eczema Area and Severity Index Score
EAT study: Enquiring About Tolerance study
eCRF: Electronic case report form
FAS: Full analysis set
HEAP trial: Hen´s Egg Allergy Prevention Trial
IgE: Immunoglobulin E
IgG4: Immunoglobulin G4
LEAP trial: Learning Early About Peanut trial
OFC: Oral food challenge
PC: Phone call
PP: Per Protocol
PRACTALL: PRACTical issus of ALLergology, Joint United States/European Union Initiative
REDCap: Research Electronic Data Capture
RNA: Ribonucleic acid
SAE: Serious adverse event
SAP: Statistical analysis plan
SCORAD: SCORing Atopic Dermatits
SPT: Skin prick test
TEFFA: Tolerance induction through early feeding to prevent food allergy in infants with eczema
TEWL: Transepidermal water loss
UK: United Kingdom
V: Visit
